# Dapsone as a Detrimental Cause of Necrotizing Fasciitis With Severe Resistant Neutropenia: A Case Report

**DOI:** 10.7759/cureus.23076

**Published:** 2022-03-11

**Authors:** Juwairiah Abdur Raheem, Arshiya Unnisa, Mohammed Iqubal

**Affiliations:** 1 Department of Internal Medicine, Deccan College of Medical Sciences, Hyderabad, IND

**Keywords:** neutropenic sepsis, dapsone, adult female acne, septic shock, organ failure from sepsis, severe neutropenia, necrotizing fasciitis, dapsone side effect

## Abstract

Dapsone, which is used for treating dermatological conditions, can lead to neutropenia. Especially, resistant neutropenia makes patients vulnerable to invasive infections, indicating a medical emergency. Febrile neutropenia secondary to dapsone intake should be treated promptly before the development of sepsis, which may lead to shock and death. In addition, necrotizing fasciitis is a severe and potentially fatal soft-tissue infection that rarely develops in healthy individuals with skin lesions. In this report, we present a case of a patient with no comorbidities who presented with necrotizing fasciitis and neutropenia with a history of dapsone intake.

## Introduction

Dapsone is a widely researched drug due to its anti-inflammatory and antimicrobial properties [1,2], used particularly in cases of refractory and unusual dermatologic conditions [3,4]. It can effectively treat various blistering skin diseases [5]. Patients who respond to dapsone have a predominant neutrophilic infiltrate in their skin [5]. In rare cases, this infiltration can cause neutropenia or agranulocytosis, with a mortality rate of 50% [6]. Dapsone is known to cause neutropenia after 8 to 10 weeks of continuous intake [6]. Therefore, these patients need to be monitored and instructed to seek urgent medical help if they develop fever. The risk of developing neutropenia is more when dapsone is administered orally, in contrast to topical administration. Necrotizing fasciitis (NF) of the head and neck is an uncommon, progressive, destructive, soft-tissue infection caused by mixed aerobic and anaerobic organisms, and has a mortality rate of 22%-100% when left untreated [7]. Herein, we report a case of right-cheek swelling and abscess in a patient with a history of dapsone intake for acne vulgaris treatment. We obtained informed consent before the study started.

## Case presentation

A 27-year-old female, with no comorbidities, presented to the emergency department with chief complaints of on and off fever and chills for one week associated with vomiting and loose stools. There is a history of upper abdominal pain, gum bleeding (two to three times), and right-sided cheek and neck swelling with pus discharge for one day, secondary to acne. She had been taking oral herbal medicines and recently dapsone for the treatment of acne vulgaris for one month. The patient was in a state of shock and admitted to the Critical Care Unit for stabilization. Table 1 lists the diagnostic findings.

**Table 1:** Blood test results of the patient CECT- Contrast-enhanced computed tomography; HRCT- High-resolution computed tomography

Blood Test	Results
C-reactive protein	12 mg/dl
Random blood glucose	>200 mg/dl
Arterial blood gas	Compensated metabolic acidosis
Complete urine examination	8-10 pus cells, 5-6 red blood cells, and albuminuria
Stool examination	Occult blood positive
Coagulation profile	Prothrombin time, 18 seconds; international normal ratio, 1.4
Thyroid-stimulating hormone	7 uIU/ml
Liver function tests	Serum albumin, 2.5 g/dl; alkaline phosphatase, 48 IU/L; alanine transaminase, 68 IU/L; total bilirubin, 5.8 mg/dl (direct, 3 mg/dl; indirect, 2.8 mg/dl)
Widal test	Negative
Antinuclear antibodies and double-stranded DNA	Negative
CECT of the Neck	Cervical lymphadenopathy
HRCT of the Chest	Atelectatic changes in the superior segment of the left lower lobe

**Figure 1:**
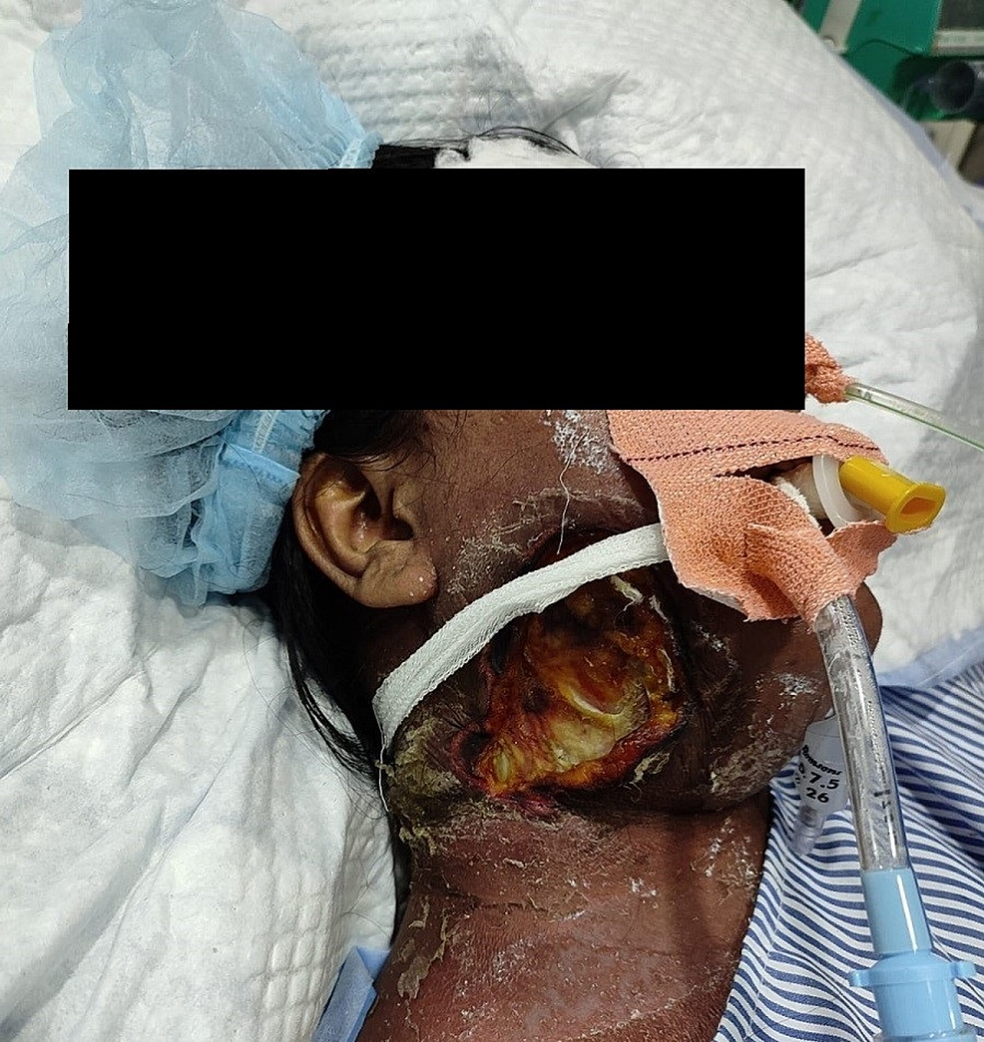
Fasciotomy performed on the right cheek

Based on the diagnostic test results, the patient was diagnosed with right-side NF of the cheek and neck with urosepsis, high-grade fever, and acute gastroenteritis. The patient underwent emergency fasciotomy in the operating theater (Figure 1).

**Figure 2:**
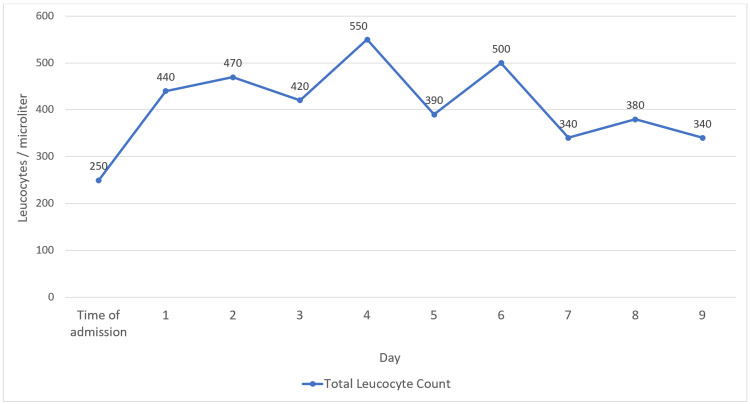
Leucocyte rate during the hospital stay

She was administered with a broad coverage of antibacterial, antiviral, antifungal agents (meropenem, acyclovir, fluconazole and teicoplanin) with antipyretics and tepid sponging. She was intubated and kept on mechanical ventilation. Subsequently, she developed pancytopenia with severe neutropenia (Figure 2) and received Granulocyte-Colony Stimulating Factor (GCSF).

However, the patient’s condition deteriorated, progressing to multiple organ failure. The case was managed by a multidisciplinary team consisting of physician, surgeon, gastroenterologist, otolaryngologist, dietician, microbiologist, and pathologist. Bicytopenia rapidly worsened, and the opinion of a hematologist oncologist was sought. The patient was kept in isolation, and a bone-marrow biopsy was planned to identify the cause of resistant neutropenia. She then developed hypoxia, and chest X-ray revealed pulmonary infiltrates in the right middle lobe. She also developed sudden bradycardia with hypotension and ultimately went into asystole. Eight cycles of cardiopulmonary resuscitation (CPR) were given according to the Advanced Cardiovascular Life Support protocol. A return of spontaneous circulation (ROSC) was achieved, with a heart rate of 73 beats/minute and a palpable pulse. However, post-CPR electrocardiography revealed supraventricular tachycardia, leading to bradycardia and cardiopulmonary arrest again. Despite adequate CPR and resuscitative measures, ROSC could no longer be achieved.

## Discussion

Neutrophils, which are produced by the bone marrow, are important for infection control, with a lower acceptable limit of 1500 neutrophils/µL of blood. A value of less than 500/µL indicates severe neutropenia, which is mostly resistant and does not respond to symptomatic treatment and GCSF. When used in the management of dermatologic conditions, dapsone (a prototype of sulphones) may cause neutropenia (a known idiosyncratic complication) [8,9]. Given the suppressed immune system, fever is usually the only presenting complaint. Febrile neutropenia causes occult infections in the body, primarily bacterial and rarely viral or fungal [10]. After receiving antimicrobials, patients could recover or suffer from life-threatening infections [10]. Patients with severe neutropenia need to be kept in neutropenic isolation as a precautionary measure to protect them from infections. Taking herbal medicines can cause drug interactions, hepatic failure, and renal failure, eventually progressing to multiple organ dysfunction syndrome (MODS). Our patient presented with high-grade fever, severe neutropenia, and NF (secondary to dapsone), causing septic shock and death despite adequate treatment and strict isolation measures.

NF is a rare, life-threatening, rapidly progressive inflammatory infection, with secondary necrosis of the subcutaneous tissues. It progresses in an insidious manner, and by the time it is diagnosed, the condition is already in the late stage. In the early stages, patients may have a warm and reddish skin and present with flu-like symptoms. NF needs to be managed immediately by incision and drainage and broad antimicrobial coverage because it can lead to sepsis progressing to shock and multiple organ failure. Neutropenic sepsis (NS) is a medical emergency because it makes patients susceptible to invasive infections, rapidly progressing to septic shock and death [11]. Even without the typical features of sepsis, NS should be diagnosed early and treated promptly. Measures to tackle NS include intensive supportive management as the standard of care [12]. Patients should be treated by administering intravenous fluids, broad-spectrum antibacterial, antiviral, and antifungals. When neutropenia is suspected, treatment should be initiated and not be delayed until the condition is confirmed. Workup on resistant neutropenia should include bone-marrow biopsy. Furthermore, sepsis-associated cardiac arrest is relatively common and leads to a poorer outcome [13], where CPR might no longer be successful.

## Conclusions

Concurrent intake of dapsone and herbal medicines may cause resistant febrile neutropenia and NF, resulting in sepsis and MODS if not treated promptly. However, the exact cause of disease manifestation in our patient, who was a young female with no comorbidities and no known cause of NF, remains elusive. Overall, febrile neutropenia is still difficult to treat, where patients experience severe sepsis and do not respond to a wide coverage of antibacterial, antiviral, and antifungal treatments and isolation.
